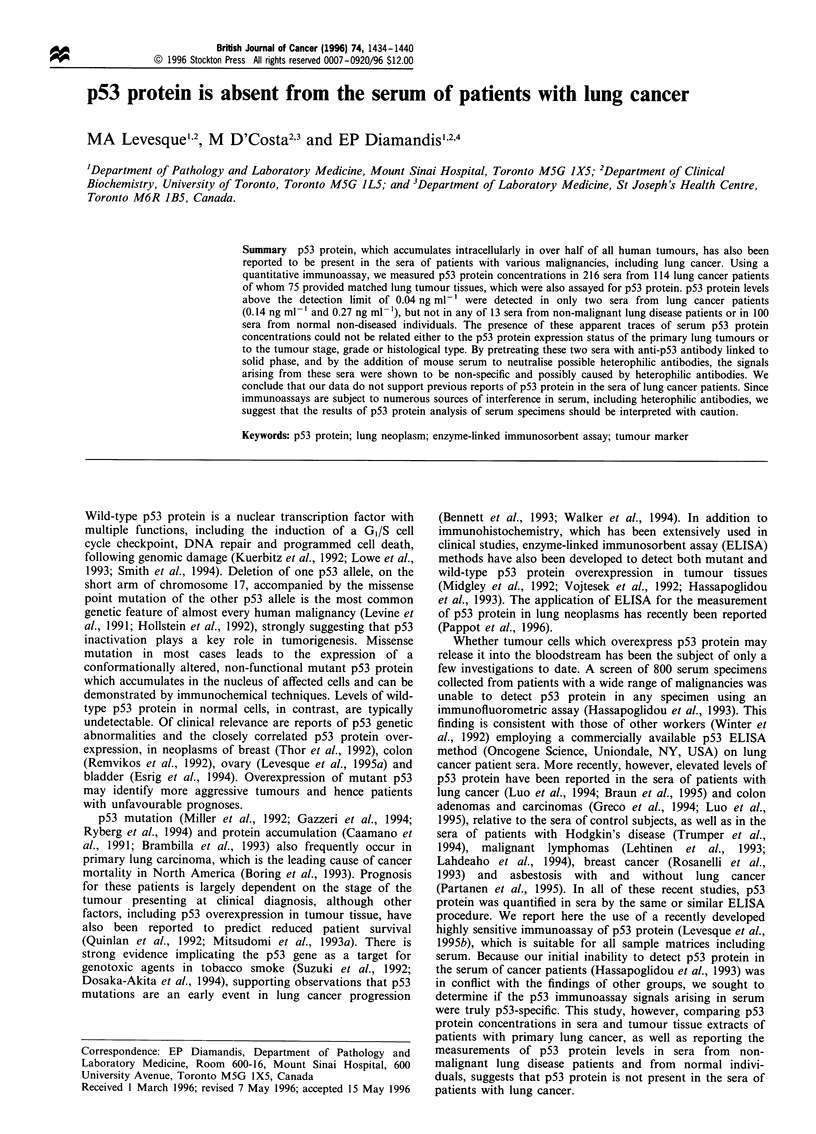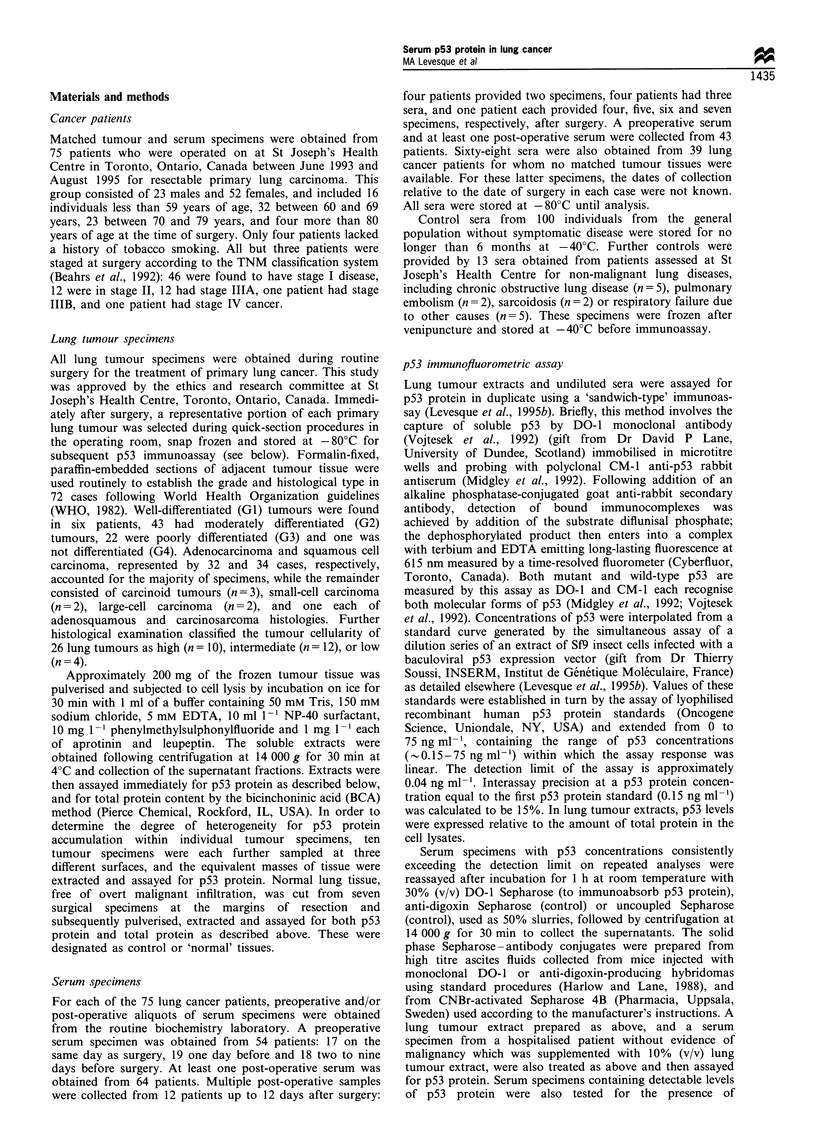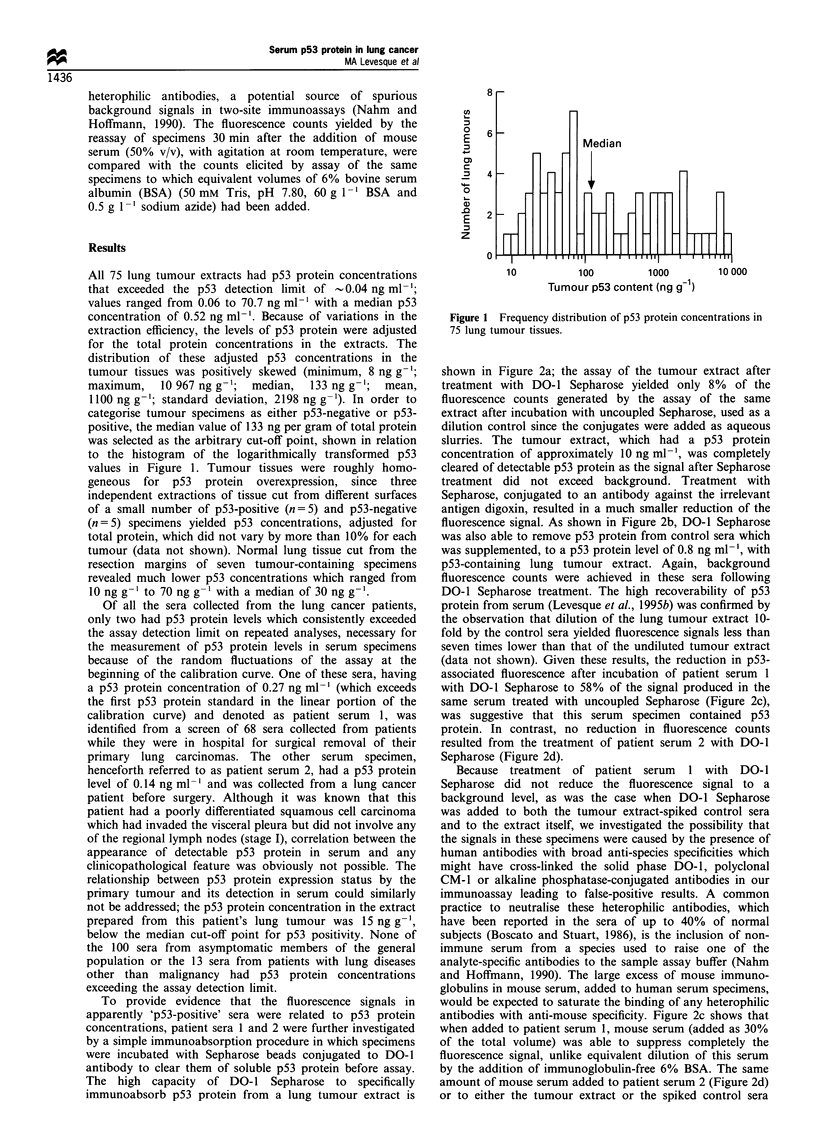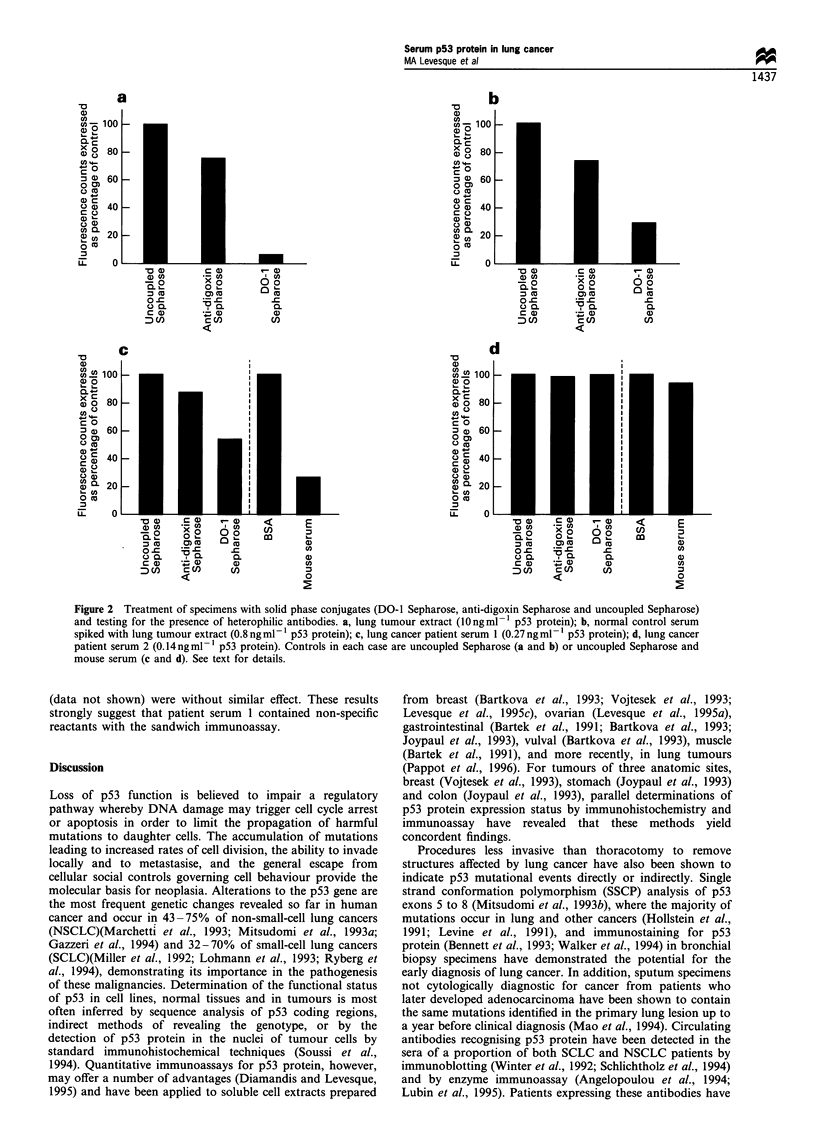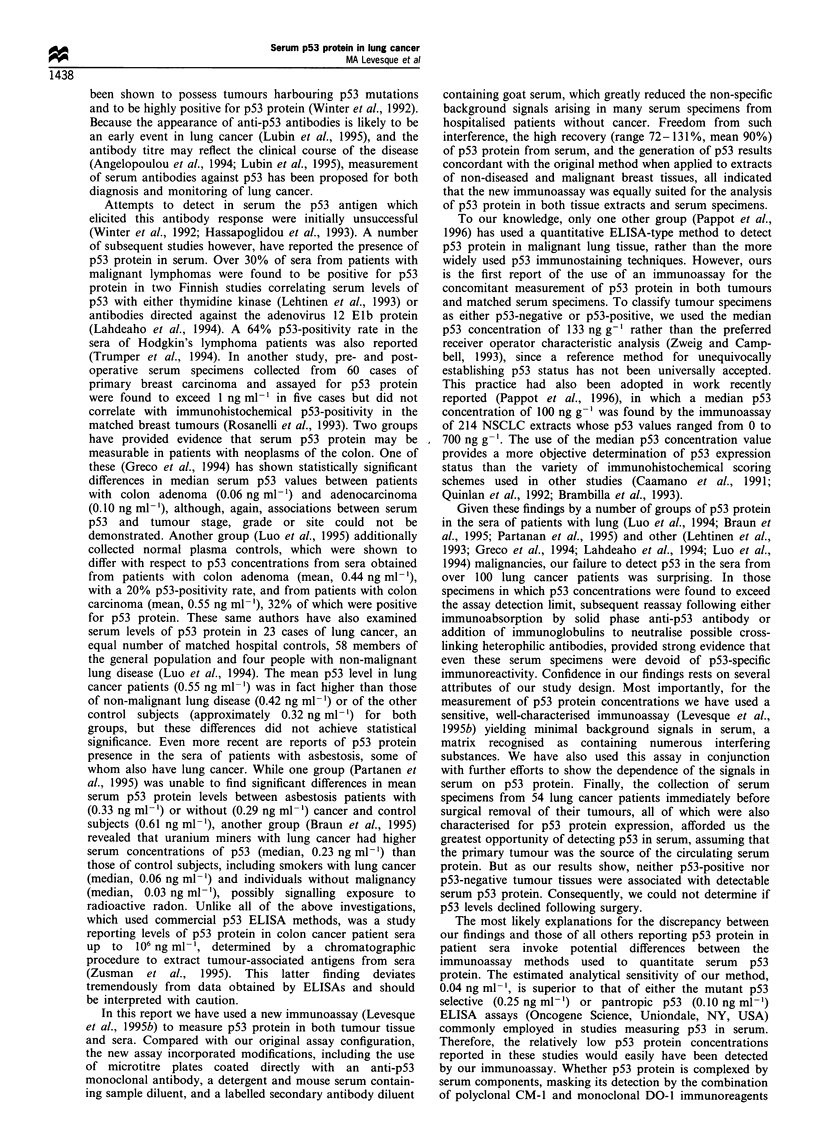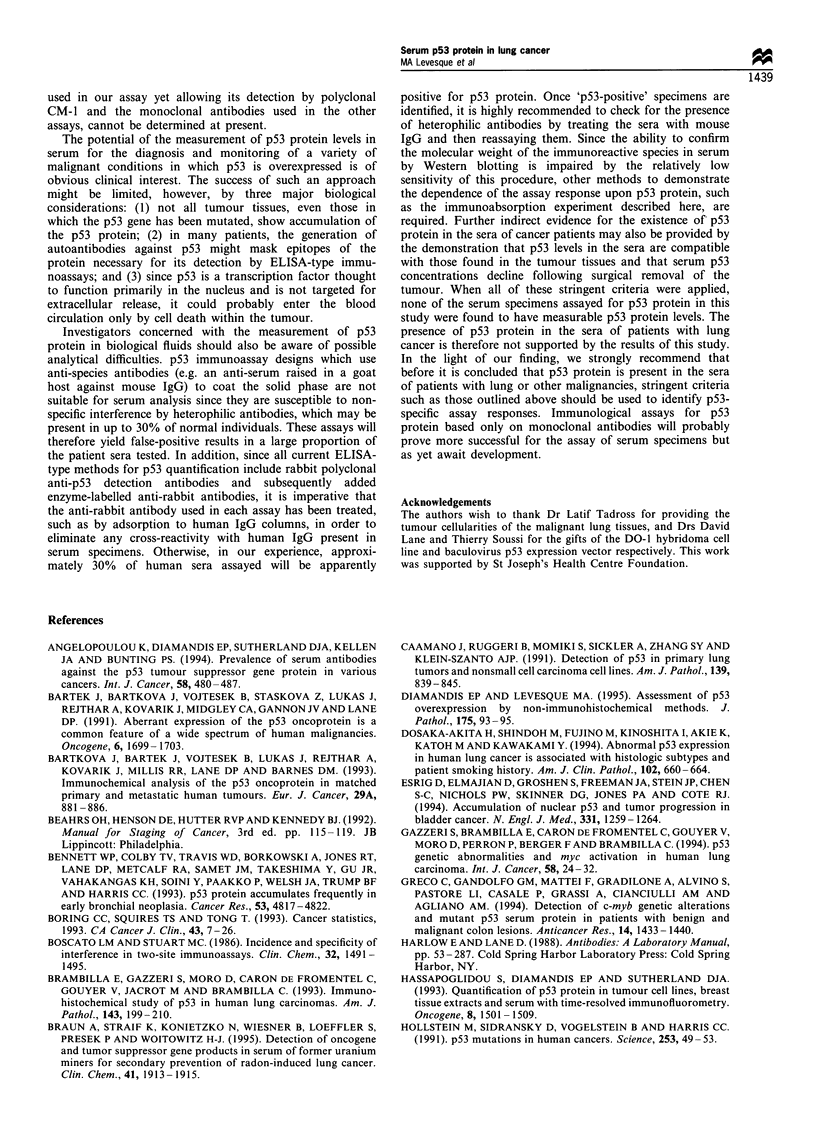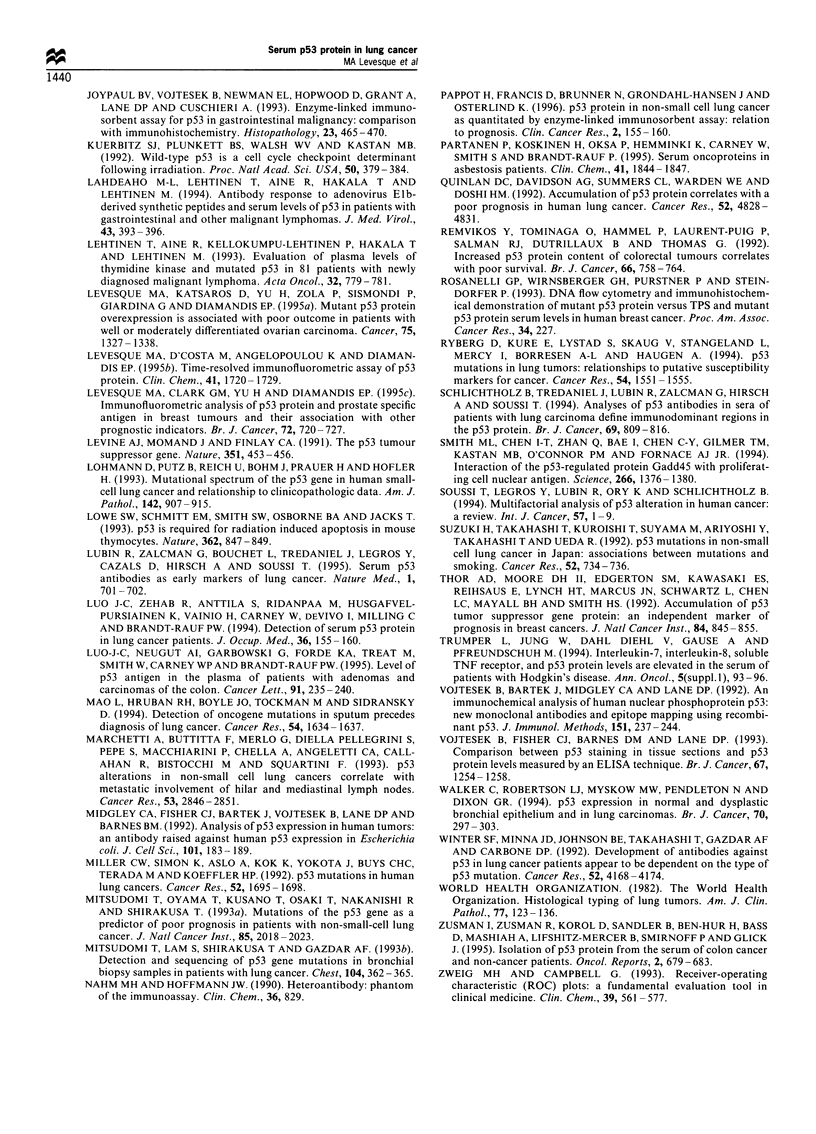# p53 protein is absent from the serum of patients with lung cancer.

**DOI:** 10.1038/bjc.1996.561

**Published:** 1996-11

**Authors:** M. A. Levesque, M. D'Costa, E. P. Diamandis

**Affiliations:** Department of Pathology, Mount Sinai Hospital, Toronto, Canada.

## Abstract

p53 protein, which accumulates intracellularly in over half of all human tumours, has also been reported to be present in the sera of patients with various malignancies, including lung cancer. Using a quantitative immunoassay, we measured p53 protein concentrations in 216 sera from 114 lung cancer patients of whom 75 provided matched lung tumour tissues, which were also assayed for p53 protein. p53 protein levels above the detection limit of 0.04 ng ml-1 were detected in only two sera from lung cancer patients (0.14 ng ml-1 and 0.27 ng ml-1), but not in any of 13 sera from non-malignant lung disease patients or in 100 sera from normal non-diseased individuals. The presence of these apparent traces of serum p53 protein concentrations could not be related either to the p53 protein expression status of the primary lung tumours or to the tumour stage, grade or histological type. By pretreating these two sera with anti-p53 antibody linked to solid phase, and by the addition of mouse serum to neutralise possible heterophilic antibodies, the signals arising from these sera were shown to be non-specific and possibly caused by heterophilic antibodies. We conclude that our data do not support previous reports of p53 protein in the sera of lung cancer patients. Since immunoassays are subject to numerous sources of interference in serum, including heterophilic antibodies, we suggest that the results of p53 protein analysis of serum specimens should be interpreted with caution.


British Journal of Cancer (1996) 74, 1434–1440
© 1996 Stockton Press All rights reserved 0007–0920/96 $12.00

### p53 protein is absent from the serum of patients with lung cancer

MA Levesque[1,2], M D'Costa[2,3] and EP Diamandis[1,2,4]

[1]*Department of Pathology and Laboratory Medicine, Mount Sinai Hospital, Toronto M5G 1X5;* [2]*Department of Clinical Biochemistry, University of Toronto, Toronto M5G 1L5; and* [3]*Department of Laboratory Medicine, St Joseph's Health Centre, Toronto M6R 1B5, Canada.*

**Summary** p53 protein, which accumulates intracellularly in over half of all human tumours, has also been reported to be present in the sera of patients with various malignancies, including lung cancer. Using a quantitative immunoassay, we measured p53 protein concentrations in 216 sera from 114 lung cancer patients of whom 75 provided matched lung tumour tissues, which were also assayed for p53 protein. p53 protein levels above the detection limit of 0.04 ng $ml^{-1}$ were detected in only two sera from lung cancer patients (0.14 ng $ml^{-1}$ and 0.27 ng $ml^{-1}$), but not in any of 13 sera from non-malignant lung disease patients or in 100 sera from normal non-diseased individuals. The presence of these apparent traces of serum p53 protein concentrations could not be related either to the p53 protein expression status of the primary lung tumours or to the tumour stage, grade or histological type. By pretreating these two sera with anti-p53 antibody linked to solid phase, and by the addition of mouse serum to neutralise possible heterophilic antibodies, the signals arising from these sera were shown to be non-specific and possibly caused by heterophilic antibodies. We conclude that our data do not support previous reports of p53 protein in the sera of lung cancer patients. Since immunoassays are subject to numerous sources of interference in serum, including heterophilic antibodies, we suggest that the results of p53 protein analysis of serum specimens should be interpreted with caution.

**Keywords:** p53 protein; lung neoplasm; enzyme-linked immunosorbent assay; tumour marker

Wild-type p53 protein is a nuclear transcription factor with multiple functions, including the induction of a $G_1/S$ cell cycle checkpoint, DNA repair and programmed cell death, following genomic damage (Kuerbitz *et al.*, 1992; Lowe *et al.*, 1993; Smith *et al.*, 1994). Deletion of one p53 allele, on the short arm of chromosome 17, accompanied by the missense point mutation of the other p53 allele is the most common genetic feature of almost every human malignancy (Levine *et al.*, 1991; Hollstein *et al.*, 1992), strongly suggesting that p53 inactivation plays a key role in tumorigenesis. Missense mutation in most cases leads to the expression of a conformationally altered, non-functional mutant p53 protein which accumulates in the nucleus of affected cells and can be demonstrated by immunochemical techniques. Levels of wild-type p53 protein in normal cells, in contrast, are typically undetectable. Of clinical relevance are reports of p53 genetic abnormalities and the closely correlated p53 protein overexpression, in neoplasms of breast (Thor *et al.*, 1992), colon (Remvikos *et al.*, 1992), ovary (Levesque *et al.*, 1995*a*) and bladder (Esrig *et al.*, 1994). Overexpression of mutant p53 may identify more aggressive tumours and hence patients with unfavourable prognoses.

p53 mutation (Miller *et al.*, 1992; Gazzeri *et al.*, 1994; Ryberg *et al.*, 1994) and protein accumulation (Caamano *et al.*, 1991; Brambilla *et al.*, 1993) also frequently occur in primary lung carcinoma, which is the leading cause of cancer mortality in North America (Boring *et al.*, 1993). Prognosis for these patients is largely dependent on the stage of the tumour presenting at clinical diagnosis, although other factors, including p53 overexpression in tumour tissue, have also been reported to predict reduced patient survival (Quinlan *et al.*, 1992; Mitsudomi *et al.*, 1993*a*). There is strong evidence implicating the p53 gene as a target for genotoxic agents in tobacco smoke (Suzuki *et al.*, 1992; Dosaka-Akita *et al.*, 1994), supporting observations that p53 mutations are an early event in lung cancer progression (Bennett *et al.*, 1993; Walker *et al.*, 1994). In addition to immunohistochemistry, which has been extensively used in clinical studies, enzyme-linked immunosorbent assay (ELISA) methods have also been developed to detect both mutant and wild-type p53 protein overexpression in tumour tissues (Midgley *et al.*, 1992; Vojtesek *et al.*, 1992; Hassapoglidou *et al.*, 1993). The application of ELISA for the measurement of p53 protein in lung neoplasms has recently been reported (Pappot *et al.*, 1996).

Whether tumour cells which overexpress p53 protein may release it into the bloodstream has been the subject of only a few investigations to date. A screen of 800 serum specimens collected from patients with a wide range of malignancies was unable to detect p53 protein in any specimen using an immunofluorometric assay (Hassapoglidou *et al.*, 1993). This finding is consistent with those of other workers (Winter *et al.*, 1992) employing a commercially available p53 ELISA method (Oncogene Science, Uniondale, NY, USA) on lung cancer patient sera. More recently, however, elevated levels of p53 protein have been reported in the sera of patients with lung cancer (Luo *et al.*, 1994; Braun *et al.*, 1995) and colon adenomas and carcinomas (Greco *et al.*, 1994; Luo *et al.*, 1995), relative to the sera of control subjects, as well as in the sera of patients with Hodgkin's disease (Trumper *et al.*, 1994), malignant lymphomas (Lehtinen *et al.*, 1993; Lahdeaho *et al.*, 1994), breast cancer (Rosanelli *et al.*, 1993) and asbestosis with and without lung cancer (Partanen *et al.*, 1995). In all of these recent studies, p53 protein was quantified in sera by the same or similar ELISA procedure. We report here the use of a recently developed highly sensitive immunoassay of p53 protein (Levesque *et al.*, 1995*b*), which is suitable for all sample matrices including serum. Because our initial inability to detect p53 protein in the serum of cancer patients (Hassapoglidou *et al.*, 1993) was in conflict with the findings of other groups, we sought to determine if the p53 immunoassay signals arising in serum were truly p53-specific. This study, however, comparing p53 protein concentrations in sera and tumour tissue extracts of patients with primary lung cancer, as well as reporting the measurements of p53 protein levels in sera from non-malignant lung disease patients and from normal individuals, suggests that p53 protein is not present in the sera of patients with lung cancer.

Correspondence: EP Diamandis, Department of Pathology and Laboratory Medicine, Room 600-16, Mount Sinai Hospital, 600 University Avenue, Toronto M5G 1X5, Canada
Received 1 March 1996; revised 7 May 1996; accepted 15 May 1996

### Materials and methods

*Cancer patients*

Matched tumour and serum specimens were obtained from 75 patients who were operated on at St Joseph's Health Centre in Toronto, Ontario, Canada between June 1993 and August 1995 for resectable primary lung carcinoma. This group consisted of 23 males and 52 females, and included 16 individuals less than 59 years of age, 32 between 60 and 69 years, 23 between 70 and 79 years, and four more than 80 years of age at the time of surgery. Only four patients lacked a history of tobacco smoking. All but three patients were staged at surgery according to the TNM classification system (Beahrs *et al.*, 1992): 46 were found to have stage I disease, 12 were in stage II, 12 had stage IIIA, one patient had stage IIIB, and one patient had stage IV cancer.

*Lung tumour specimens*

All lung tumour specimens were obtained during routine surgery for the treatment of primary lung cancer. This study was approved by the ethics and research committee at St Joseph's Health Centre, Toronto, Ontario, Canada. Immediately after surgery, a representative portion of each primary lung tumour was selected during quick-section procedures in the operating room, snap frozen and stored at $-80°C$ for subsequent p53 immunoassay (see below). Formalin-fixed, paraffin-embedded sections of adjacent tumour tissue were used routinely to establish the grade and histological type in 72 cases following World Health Organization guidelines (WHO, 1982). Well-differentiated (G1) tumours were found in six patients, 43 had moderately differentiated (G2) tumours, 22 were poorly differentiated (G3) and one was not differentiated (G4). Adenocarcinoma and squamous cell carcinoma, represented by 32 and 34 cases, respectively, accounted for the majority of specimens, while the remainder consisted of carcinoid tumours ($n=3$), small-cell carcinoma ($n=2$), large-cell carcinoma ($n=2$), and one each of adenosquamous and carcinosarcoma histologies. Further histological examination classified the tumour cellularity of 26 lung tumours as high ($n=10$), intermediate ($n=12$), or low ($n=4$).

Approximately 200 mg of the frozen tumour tissue was pulverised and subjected to cell lysis by incubation on ice for 30 min with 1 ml of a buffer containing 50 mM Tris, 150 mM sodium chloride, 5 mM EDTA, 10 ml $l^{-1}$ NP-40 surfactant, 10 mg $l^{-1}$ phenylmethylsulphonylfluoride and 1 mg $l^{-1}$ each of aprotinin and leupeptin. The soluble extracts were obtained following centrifugation at 14 000 $g$ for 30 min at 4°C and collection of the supernatant fractions. Extracts were then assayed immediately for p53 protein as described below, and for total protein content by the bicinchoninic acid (BCA) method (Pierce Chemical, Rockford, IL, USA). In order to determine the degree of heterogeneity for p53 protein accumulation within individual tumour specimens, ten tumour specimens were each further sampled at three different surfaces, and the equivalent masses of tissue were extracted and assayed for p53 protein. Normal lung tissue, free of overt malignant infiltration, was cut from seven surgical specimens at the margins of resection and subsequently pulverised, extracted and assayed for both p53 protein and total protein as described above. These were designated as control or 'normal' tissues.

*Serum specimens*

For each of the 75 lung cancer patients, preoperative and/or post-operative aliquots of serum specimens were obtained from the routine biochemistry laboratory. A preoperative serum specimen was obtained from 54 patients: 17 on the same day as surgery, 19 one day before and 18 two to nine days before surgery. At least one post-operative serum was obtained from 64 patients. Multiple post-operative samples were collected from 12 patients up to 12 days after surgery: four patients provided two specimens, four patients had three sera, and one patient each provided four, five, six and seven specimens, respectively, after surgery. A preoperative serum and at least one post-operative serum were collected from 43 patients. Sixty-eight sera were also obtained from 39 lung cancer patients for whom no matched tumour tissues were available. For these latter specimens, the dates of collection relative to the date of surgery in each case were not known. All sera were stored at $-80°C$ until analysis.

Control sera from 100 individuals from the general population without symptomatic disease were stored for no longer than 6 months at $-40°C$. Further controls were provided by 13 sera obtained from patients assessed at St Joseph's Health Centre for non-malignant lung diseases, including chronic obstructive lung disease ($n=5$), pulmonary embolism ($n=2$), sarcoidosis ($n=2$) or respiratory failure due to other causes ($n=5$). These specimens were frozen after venipuncture and stored at $-40°C$ before immunoassay.

*p53 immunofluorometric assay*

Lung tumour extracts and undiluted sera were assayed for p53 protein in duplicate using a 'sandwich-type' immunoassay (Levesque *et al.*, 1995*b*). Briefly, this method involves the capture of soluble p53 by DO-1 monoclonal antibody (Vojtesek *et al.*, 1992) (gift from Dr David P Lane, University of Dundee, Scotland) immobilised in microtitre wells and probing with polyclonal CM-1 anti-p53 rabbit antiserum (Midgley *et al.*, 1992). Following addition of an alkaline phosphatase-conjugated goat anti-rabbit secondary antibody, detection of bound immunocomplexes was achieved by addition of the substrate diflunisal phosphate; the dephosphorylated product then enters into a complex with terbium and EDTA emitting long-lasting fluorescence at 615 nm measured by a time-resolved fluorometer (Cyberfluor, Toronto, Canada). Both mutant and wild-type p53 are measured by this assay as DO-1 and CM-1 each recognise both molecular forms of p53 (Midgley *et al.*, 1992; Vojtesek *et al.*, 1992). Concentrations of p53 were interpolated from a standard curve generated by the simultaneous assay of a dilution series of an extract of Sf9 insect cells infected with a baculoviral p53 expression vector (gift from Dr Thierry Soussi, INSERM, Institut de Génétique Moléculaire, France) as detailed elsewhere (Levesque *et al.*, 1995*b*). Values of these standards were established in turn by the assay of lyophilised recombinant human p53 protein standards (Oncogene Science, Uniondale, NY, USA) and extended from 0 to 75 ng $ml^{-1}$, containing the range of p53 concentrations ($\sim$0.15–75 ng $ml^{-1}$) within which the assay response was linear. The detection limit of the assay is approximately 0.04 ng $ml^{-1}$. Interassay precision at a p53 protein concentration equal to the first p53 protein standard (0.15 ng $ml^{-1}$) was calculated to be 15%. In lung tumour extracts, p53 levels were expressed relative to the amount of total protein in the cell lysates.

Serum specimens with p53 concentrations consistently exceeding the detection limit on repeated analyses were reassayed after incubation for 1 h at room temperature with 30% (v/v) DO-1 Sepharose (to immunoabsorb p53 protein), anti-digoxin Sepharose (control) or uncoupled Sepharose (control), used as 50% slurries, followed by centrifugation at 14 000 $g$ for 30 min to collect the supernatants. The solid phase Sepharose–antibody conjugates were prepared from high titre ascites fluids collected from mice injected with monoclonal DO-1 or anti-digoxin-producing hybridomas using standard procedures (Harlow and Lane, 1988), and from CNBr-activated Sepharose 4B (Pharmacia, Uppsala, Sweden) used according to the manufacturer's instructions. A lung tumour extract prepared as above, and a serum specimen from a hospitalised patient without evidence of malignancy which was supplemented with 10% (v/v) lung tumour extract, were also treated as above and then assayed for p53 protein. Serum specimens containing detectable levels of p53 protein were also tested for the presence of heterophilic antibodies, a potential source of spurious background signals in two-site immunoassays (Nahm and Hoffmann, 1990). The fluorescence counts yielded by the reassay of specimens 30 min after the addition of mouse serum (50% v/v), with agitation at room temperature, were compared with the counts elicited by assay of the same specimens to which equivalent volumes of 6% bovine serum albumin (BSA) (50 mM Tris, pH 7.80, 60 g $l^{-1}$ BSA and 0.5 g $l^{-1}$ sodium azide) had been added.

### Results

All 75 lung tumour extracts had p53 protein concentrations that exceeded the p53 detection limit of ~0.04 ng $ml^{-1}$; values ranged from 0.06 to 70.7 ng $ml^{-1}$ with a median p53 concentration of 0.52 ng $ml^{-1}$. Because of variations in the extraction efficiency, the levels of p53 protein were adjusted for the total protein concentrations in the extracts. The distribution of these adjusted p53 concentrations in the tumour tissues was positively skewed (minimum, 8 ng $g^{-1}$; maximum, 10 967 ng $g^{-1}$; median, 133 ng $g^{-1}$; mean, 1100 ng $g^{-1}$; standard deviation, 2198 ng $g^{-1}$). In order to categorise tumour specimens as either p53-negative or p53-positive, the median value of 133 ng per gram of total protein was selected as the arbitrary cut-off point, shown in relation to the histogram of the logarithmically transformed p53 values in Figure 1. Tumour tissues were roughly homogeneous for p53 protein overexpression, since three independent extractions of tissue cut from different surfaces of a small number of p53-positive ($n=5$) and p53-negative ($n=5$) specimens yielded p53 concentrations, adjusted for total protein, which did not vary by more than 10% for each tumour (data not shown). Normal lung tissue cut from the resection margins of seven tumour-containing specimens revealed much lower p53 concentrations which ranged from 10 ng $g^{-1}$ to 70 ng $g^{-1}$ with a median of 30 ng $g^{-1}$.

Of all the sera collected from the lung cancer patients, only two had p53 protein levels which consistently exceeded the assay detection limit on repeated analyses, necessary for the measurement of p53 protein levels in serum specimens because of the random fluctuations of the assay at the beginning of the calibration curve. One of these sera, having a p53 protein concentration of 0.27 ng $ml^{-1}$ (which exceeds the first p53 protein standard in the linear portion of the calibration curve) and denoted as patient serum 1, was identified from a screen of 68 sera collected from patients while they were in hospital for surgical removal of their primary lung carcinomas. The other serum specimen, henceforth referred to as patient serum 2, had a p53 protein level of 0.14 ng $ml^{-1}$ and was collected from a lung cancer patient before surgery. Although it was known that this patient had a poorly differentiated squamous cell carcinoma which had invaded the visceral pleura but did not involve any of the regional lymph nodes (stage I), correlation between the appearance of detectable p53 protein in serum and any clinicopathological feature was obviously not possible. The relationship between p53 protein expression status by the primary tumour and its detection in serum could similarly not be addressed; the p53 protein concentration in the extract prepared from this patient's lung tumour was 15 ng $g^{-1}$, below the median cut-off point for p53 positivity. None of the 100 sera from asymptomatic members of the general population or the 13 sera from patients with lung diseases other than malignancy had p53 protein concentrations exceeding the assay detection limit.

To provide evidence that the fluorescence signals in apparently 'p53-positive' sera were related to p53 protein concentrations, patient sera 1 and 2 were further investigated by a simple immunoabsorption procedure in which specimens were incubated with Sepharose beads conjugated to DO-1 antibody to clear them of soluble p53 protein before assay. The high capacity of DO-1 Sepharose to specifically immunoabsorb p53 protein from a lung tumour extract is

**Figure 1** Frequency distribution of p53 protein concentrations in 75 lung tumour tissues.

shown in Figure 2a; the assay of the tumour extract after treatment with DO-1 Sepharose yielded only 8% of the fluorescence counts generated by the assay of the same extract after incubation with uncoupled Sepharose, used as a dilution control since the conjugates were added as aqueous slurries. The tumour extract, which had a p53 protein concentration of approximately 10 ng $ml^{-1}$, was completely cleared of detectable p53 protein as the signal after Sepharose treatment did not exceed background. Treatment with Sepharose, conjugated to an antibody against the irrelevant antigen digoxin, resulted in a much smaller reduction of the fluorescence signal. As shown in Figure 2b, DO-1 Sepharose was also able to remove p53 protein from control sera which was supplemented, to a p53 protein level of 0.8 ng $ml^{-1}$, with p53-containing lung tumour extract. Again, background fluorescence counts were achieved in these sera following DO-1 Sepharose treatment. The high recoverability of p53 protein from serum (Levesque *et al.*, 1995*b*) was confirmed by the observation that dilution of the lung tumour extract 10-fold by the control sera yielded fluorescence signals less than seven times lower than that of the undiluted tumour extract (data not shown). Given these results, the reduction in p53-associated fluorescence after incubation of patient serum 1 with DO-1 Sepharose to 58% of the signal produced in the same serum treated with uncoupled Sepharose (Figure 2c), was suggestive that this serum specimen contained p53 protein. In contrast, no reduction in fluorescence counts resulted from the treatment of patient serum 2 with DO-1 Sepharose (Figure 2d).

Because treatment of patient serum 1 with DO-1 Sepharose did not reduce the fluorescence signal to a background level, as was the case when DO-1 Sepharose was added to both the tumour extract-spiked control sera and to the extract itself, we investigated the possibility that the signals in these specimens were caused by the presence of human antibodies with broad anti-species specificities which might have cross-linked the solid phase DO-1, polyclonal CM-1 or alkaline phosphatase-conjugated antibodies in our immunoassay leading to false-positive results. A common practice to neutralise these heterophilic antibodies, which have been reported in the sera of up to 40% of normal subjects (Boscato and Stuart, 1986), is the inclusion of non-immune serum from a species used to raise one of the analyte-specific antibodies to the sample assay buffer (Nahm and Hoffmann, 1990). The large excess of mouse immunoglobulins in mouse serum, added to human serum specimens, would be expected to saturate the binding of any heterophilic antibodies with anti-mouse specificity. Figure 2c shows that when added to patient serum 1, mouse serum (added as 30% of the total volume) was able to suppress completely the fluorescence signal, unlike equivalent dilution of this serum by the addition of immunoglobulin-free 6% BSA. The same amount of mouse serum added to patient serum 2 (Figure 2d) or to either the tumour extract or the spiked control sera

**Figure 2** Treatment of specimens with solid phase conjugates (DO-1 Sepharose, anti-digoxin Sepharose and uncoupled Sepharose) and testing for the presence of heterophilic antibodies. **a**, lung tumour extract (10 ng ml$^{-1}$ p53 protein); **b**, normal control serum spiked with lung tumour extract (0.8 ng ml$^{-1}$ p53 protein); **c**, lung cancer patient serum 1 (0.27 ng ml$^{-1}$ p53 protein); **d**, lung cancer patient serum 2 (0.14 ng ml$^{-1}$ p53 protein). Controls in each case are uncoupled Sepharose (**a** and **b**) or uncoupled Sepharose and mouse serum (**c** and **d**). See text for details.

(data not shown) were without similar effect. These results strongly suggest that patient serum 1 contained non-specific reactants with the sandwich immunoassay.

### Discussion

Loss of p53 function is believed to impair a regulatory pathway whereby DNA damage may trigger cell cycle arrest or apoptosis in order to limit the propagation of harmful mutations to daughter cells. The accumulation of mutations leading to increased rates of cell division, the ability to invade locally and to metastasise, and the general escape from cellular social controls governing cell behaviour provide the molecular basis for neoplasia. Alterations to the p53 gene are the most frequent genetic changes revealed so far in human cancer and occur in 43–75% of non-small-cell lung cancers (NSCLC)(Marchetti *et al.*, 1993; Mitsudomi *et al.*, 1993*a*; Gazzeri *et al.*, 1994) and 32–70% of small-cell lung cancers (SCLC)(Miller *et al.*, 1992; Lohmann *et al.*, 1993; Ryberg *et al.*, 1994), demonstrating its importance in the pathogenesis of these malignancies. Determination of the functional status of p53 in cell lines, normal tissues and in tumours is most often inferred by sequence analysis of p53 coding regions, indirect methods of revealing the genotype, or by the detection of p53 protein in the nuclei of tumour cells by standard immunohistochemical techniques (Soussi *et al.*, 1994). Quantitative immunoassays for p53 protein, however, may offer a number of advantages (Diamandis and Levesque, 1995) and have been applied to soluble cell extracts prepared from breast (Bartkova *et al.*, 1993; Vojtesek *et al.*, 1993; Levesque *et al.*, 1995*c*), ovarian (Levesque *et al.*, 1995*a*), gastrointestinal (Bartek *et al.*, 1991; Bartkova *et al.*, 1993; Joypaul *et al.*, 1993), vulval (Bartkova *et al.*, 1993), muscle (Bartek *et al.*, 1991), and more recently, in lung tumours (Pappot *et al.*, 1996). For tumours of three anatomic sites, breast (Vojtesek *et al.*, 1993), stomach (Joypaul *et al.*, 1993) and colon (Joypaul *et al.*, 1993), parallel determinations of p53 protein expression status by immunohistochemistry and immunoassay have revealed that these methods yield concordent findings.

Procedures less invasive than thoracotomy to remove structures affected by lung cancer have also been shown to indicate p53 mutational events directly or indirectly. Single strand conformation polymorphism (SSCP) analysis of p53 exons 5 to 8 (Mitsudomi *et al.*, 1993*b*), where the majority of mutations occur in lung and other cancers (Hollstein *et al.*, 1991; Levine *et al.*, 1991), and immunostaining for p53 protein (Bennett *et al.*, 1993; Walker *et al.*, 1994) in bronchial biopsy specimens have demonstrated the potential for the early diagnosis of lung cancer. In addition, sputum specimens not cytologically diagnostic for cancer from patients who later developed adenocarcinoma have been shown to contain the same mutations identified in the primary lung lesion up to a year before clinical diagnosis (Mao *et al.*, 1994). Circulating antibodies recognising p53 protein have been detected in the sera of a proportion of both SCLC and NSCLC patients by immunoblotting (Winter *et al.*, 1992; Schlichtholz *et al.*, 1994) and by enzyme immunoassay (Angelopoulou *et al.*, 1994; Lubin *et al.*, 1995). Patients expressing these antibodies have been shown to possess tumours harbouring p53 mutations and to be highly positive for p53 protein (Winter et al., 1992). Because the appearance of anti-p53 antibodies is likely to be an early event in lung cancer (Lubin et al., 1995), and the antibody titre may reflect the clinical course of the disease (Angelopoulou et al., 1994; Lubin et al., 1995), measurement of serum antibodies against p53 has been proposed for both diagnosis and monitoring of lung cancer.

Attempts to detect in serum the p53 antigen which elicited this antibody response were initially unsuccessful (Winter et al., 1992; Hassapoglidou et al., 1993). A number of subsequent studies however, have reported the presence of p53 protein in serum. Over 30% of sera from patients with malignant lymphomas were found to be positive for p53 protein in two Finnish studies correlating serum levels of p53 with either thymidine kinase (Lehtinen et al., 1993) or antibodies directed against the adenovirus 12 Elb protein (Lahdeaho et al., 1994). A 64% p53-positivity rate in the sera of Hodgkin's lymphoma patients was also reported (Trumper et al., 1994). In another study, pre- and post-operative serum specimens collected from 60 cases of primary breast carcinoma and assayed for p53 protein were found to exceed 1 ng $ml^{-1}$ in five cases but did not correlate with immunohistochemical p53-positivity in the matched breast tumours (Rosanelli et al., 1993). Two groups have provided evidence that serum p53 protein may be measurable in patients with neoplasms of the colon. One of these (Greco et al., 1994) has shown statistically significant differences in median serum p53 values between patients with colon adenoma (0.06 ng $ml^{-1}$) and adenocarcinoma (0.10 ng $ml^{-1}$), although, again, associations between serum p53 and tumour stage, grade or site could not be demonstrated. Another group (Luo et al., 1995) additionally collected normal plasma controls, which were shown to differ with respect to p53 concentrations from sera obtained from patients with colon adenoma (mean, 0.44 ng $ml^{-1}$), with a 20% p53-positivity rate, and from patients with colon carcinoma (mean, 0.55 ng $ml^{-1}$), 32% of which were positive for p53 protein. These same authors have also examined serum levels of p53 protein in 23 cases of lung cancer, an equal number of matched hospital controls, 58 members of the general population and four people with non-malignant lung disease (Luo et al., 1994). The mean p53 level in lung cancer patients (0.55 ng $ml^{-1}$) was in fact higher than those of non-malignant lung disease (0.42 ng $ml^{-1}$) or of the other control subjects (approximately 0.32 ng $ml^{-1}$) for both groups, but these differences did not achieve statistical significance. Even more recent are reports of p53 protein presence in the sera of patients with asbestosis, some of whom also have lung cancer. While one group (Partanen et al., 1995) was unable to find significant differences in mean serum p53 protein levels between asbestosis patients with (0.33 ng $ml^{-1}$) or without (0.29 ng $ml^{-1}$) cancer and control subjects (0.61 ng $ml^{-1}$), another group (Braun et al., 1995) revealed that uranium miners with lung cancer had higher serum concentrations of p53 (median, 0.23 ng $ml^{-1}$) than those of control subjects, including smokers with lung cancer (median, 0.06 ng $ml^{-1}$) and individuals without malignancy (median, 0.03 ng $ml^{-1}$), possibly signalling exposure to radioactive radon. Unlike all of the above investigations, which used commercial p53 ELISA methods, was a study reporting levels of p53 protein in colon cancer patient sera up to $10^6$ ng $ml^{-1}$, determined by a chromatographic procedure to extract tumour-associated antigens from sera (Zusman et al., 1995). This latter finding deviates tremendously from data obtained by ELISAs and should be interpreted with caution.

In this report we have used a new immunoassay (Levesque et al., 1995b) to measure p53 protein in both tumour tissue and sera. Compared with our original assay configuration, the new assay incorporated modifications, including the use of microtitre plates coated directly with an anti-p53 monoclonal antibody, a detergent and mouse serum containing sample diluent, and a labelled secondary antibody diluent containing goat serum, which greatly reduced the non-specific background signals arising in many serum specimens from hospitalised patients without cancer. Freedom from such interference, the high recovery (range 72–131%, mean 90%) of p53 protein from serum, and the generation of p53 results concordant with the original method when applied to extracts of non-diseased and malignant breast tissues, all indicated that the new immunoassay was equally suited for the analysis of p53 protein in both tissue extracts and serum specimens.

To our knowledge, only one other group (Pappot et al., 1996) has used a quantitative ELISA-type method to detect p53 protein in malignant lung tissue, rather than the more widely used p53 immunostaining techniques. However, ours is the first report of the use of an immunoassay for the concomitant measurement of p53 protein in both tumours and matched serum specimens. To classify tumour specimens as either p53-negative or p53-positive, we used the median p53 concentration of 133 ng $g^{-1}$ rather than the preferred receiver operator characteristic analysis (Zweig and Campbell, 1993), since a reference method for unequivocally establishing p53 status has not been universally accepted. This practice had also been adopted in work recently reported (Pappot et al., 1996), in which a median p53 concentration of 100 ng $g^{-1}$ was found by the immunoassay of 214 NSCLC extracts whose p53 values ranged from 0 to 700 ng $g^{-1}$. The use of the median p53 concentration value provides a more objective determination of p53 expression status than the variety of immunohistochemical scoring schemes used in other studies (Caamano et al., 1991; Quinlan et al., 1992; Brambilla et al., 1993).

Given these findings by a number of groups of p53 protein in the sera of patients with lung (Luo et al., 1994; Braun et al., 1995; Partanan et al., 1995) and other (Lehtinen et al., 1993; Greco et al., 1994; Lahdeaho et al., 1994; Luo et al., 1994) malignancies, our failure to detect p53 in the sera from over 100 lung cancer patients was surprising. In those specimens in which p53 concentrations were found to exceed the assay detection limit, subsequent reassay following either immunoabsorption by solid phase anti-p53 antibody or addition of immunoglobulins to neutralise possible cross-linking heterophilic antibodies, provided strong evidence that even these serum specimens were devoid of p53-specific immunoreactivity. Confidence in our findings rests on several attributes of our study design. Most importantly, for the measurement of p53 protein concentrations we have used a sensitive, well-characterised immunoassay (Levesque et al., 1995b) yielding minimal background signals in serum, a matrix recognised as containing numerous interfering substances. We have also used this assay in conjunction with further efforts to show the dependence of the signals in serum on p53 protein. Finally, the collection of serum specimens from 54 lung cancer patients immediately before surgical removal of their tumours, all of which were also characterised for p53 protein expression, afforded us the greatest opportunity of detecting p53 in serum, assuming that the primary tumour was the source of the circulating serum protein. But as our results show, neither p53-positive nor p53-negative tumour tissues were associated with detectable serum p53 protein. Consequently, we could not determine if p53 levels declined following surgery.

The most likely explanations for the discrepancy between our findings and those of all others reporting p53 protein in patient sera invoke potential differences between the immunoassay methods used to quantitate serum p53 protein. The estimated analytical sensitivity of our method, 0.04 ng $ml^{-1}$, is superior to that of either the mutant p53 selective (0.25 ng $ml^{-1}$) or pantropic p53 (0.10 ng $ml^{-1}$) ELISA assays (Oncogene Science, Uniondale, NY, USA) commonly employed in studies measuring p53 in serum. Therefore, the relatively low p53 protein concentrations reported in these studies would easily have been detected by our immunoassay. Whether p53 protein is complexed by serum components, masking its detection by the combination of polyclonal CM-1 and monoclonal DO-1 immunoreagents used in our assay yet allowing its detection by polyclonal CM-1 and the monoclonal antibodies used in the other assays, cannot be determined at present.

The potential of the measurement of p53 protein levels in serum for the diagnosis and monitoring of a variety of malignant conditions in which p53 is overexpressed is of obvious clinical interest. The success of such an approach might be limited, however, by three major biological considerations: (1) not all tumour tissues, even those in which the p53 gene has been mutated, show accumulation of the p53 protein; (2) in many patients, the generation of autoantibodies against p53 might mask epitopes of the protein necessary for its detection by ELISA-type immunoassays; and (3) since p53 is a transcription factor thought to function primarily in the nucleus and is not targeted for extracellular release, it could probably enter the blood circulation only by cell death within the tumour.

Investigators concerned with the measurement of p53 protein in biological fluids should also be aware of possible analytical difficulties. p53 immunoassay designs which use anti-species antibodies (e.g. an anti-serum raised in a goat host against mouse IgG) to coat the solid phase are not suitable for serum analysis since they are susceptible to non-specific interference by heterophilic antibodies, which may be present in up to 30% of normal individuals. These assays will therefore yield false-positive results in a large proportion of the patient sera tested. In addition, since all current ELISA-type methods for p53 quantification include rabbit polyclonal anti-p53 detection antibodies and subsequently added enzyme-labelled anti-rabbit antibodies, it is imperative that the anti-rabbit antibody used in each assay has been treated, such as by adsorption to human IgG columns, in order to eliminate any cross-reactivity with human IgG present in serum specimens. Otherwise, in our experience, approximately 30% of human sera assayed will be apparently positive for p53 protein. Once 'p53-positive' specimens are identified, it is highly recommended to check for the presence of heterophilic antibodies by treating the sera with mouse IgG and then reassaying them. Since the ability to confirm the molecular weight of the immunoreactive species in serum by Western blotting is impaired by the relatively low sensitivity of this procedure, other methods to demonstrate the dependence of the assay response upon p53 protein, such as the immunoabsorption experiment described here, are required. Further indirect evidence for the existence of p53 protein in the sera of cancer patients may also be provided by the demonstration that p53 levels in the sera are compatible with those found in the tumour tissues and that serum p53 concentrations decline following surgical removal of the tumour. When all of these stringent criteria were applied, none of the serum specimens assayed for p53 protein in this study were found to have measurable p53 protein levels. The presence of p53 protein in the sera of patients with lung cancer is therefore not supported by the results of this study. In the light of our finding, we strongly recommend that before it is concluded that p53 protein is present in the sera of patients with lung or other malignancies, stringent criteria such as those outlined above should be used to identify p53-specific assay responses. Immunological assays for p53 protein based only on monoclonal antibodies will probably prove more successful for the assay of serum specimens but as yet await development.

### Acknowledgements

The authors wish to thank Dr Latif Tadross for providing the tumour cellularities of the malignant lung tissues, and Drs David Lane and Thierry Soussi for the gifts of the DO-1 hybridoma cell line and baculovirus p53 expression vector respectively. This work was supported by St Joseph's Health Centre Foundation.

### References

ANGELOPOULOU K, DIAMANDIS EP, SUTHERLAND DJA, KELLEN JA AND BUNTING PS. (1994). Prevalence of serum antibodies against the p53 tumour suppressor gene protein in various cancers. *Int. J. Cancer*, **58**, 480–487.

BARTEK J, BARTKOVA J, VOJTESEK B, STASKOVA Z, LUKAS J, REJTHAR A, KOVARIK J, MIDGLEY CA, GANNON JV AND LANE DP. (1991). Aberrant expression of the p53 oncoprotein is a common feature of a wide spectrum of human malignancies. *Oncogene*, **6**, 1699–1703.

BARTKOVA J, BARTEK J, VOJTESEK B, LUKAS J, REJTHAR A, KOVARIK J, MILLIS RR, LANE DP AND BARNES DM. (1993). Immunochemical analysis of the p53 oncoprotein in matched primary and metastatic human tumours. *Eur. J. Cancer*, **29A**, 881–886.

BEAHRS OH, HENSON DE, HUTTER RVP AND KENNEDY BJ. (1992). *Manual for Staging of Cancer*, 3rd ed. pp. 115–119. JB Lippincott: Philadelphia.

BENNETT WP, COLBY TV, TRAVIS WD, BORKOWSKI A, JONES RT, LANE DP, METCALF RA, SAMET JM, TAKESHIMA Y, GU JR, VAHAKANGAS KH, SOINI Y, PAAKKO P, WELSH JA, TRUMP BF AND HARRIS CC. (1993). p53 protein accumulates frequently in early bronchial neoplasia. *Cancer Res.*, **53**, 4817–4822.

BORING CC, SQUIRES TS AND TONG T. (1993). Cancer statistics, 1993. *CA Cancer J. Clin.*, **43**, 7–26.

BOSCATO LM AND STUART MC. (1986). Incidence and specificity of interference in two-site immunoassays. *Clin. Chem.*, **32**, 1491–1495.

BRAMBILLA E, GAZZERI S, MORO D, CARON DE FROMENTEL C, GOUYER V, JACROT M AND BRAMBILLA C. (1993). Immunohistochemical study of p53 in human lung carcinomas. *Am. J. Pathol.*, **143**, 199–210.

BRAUN A, STRAIF K, KONIETZKO N, WIESNER B, LOEFFLER S, PRESEK P AND WOITOWITZ H-J. (1995). Detection of oncogene and tumor suppressor gene products in serum of former uranium miners for secondary prevention of radon-induced lung cancer. *Clin. Chem.*, **41**, 1913–1915.

CAAMANO J, RUGGERI B, MOMIKI S, SICKLER A, ZHANG SY AND KLEIN-SZANTO AJP. (1991). Detection of p53 in primary lung tumors and nonsmall cell carcinoma cell lines. *Am. J. Pathol.*, **139**, 839–845.

DIAMANDIS EP AND LEVESQUE MA. (1995). Assessment of p53 overexpression by non-immunohistochemical methods. *J. Pathol.*, **175**, 93–95.

DOSAKA-AKITA H, SHINDOH M, FUJINO M, KINOSHITA I, AKIE K, KATOH M AND KAWAKAMI Y. (1994). Abnormal p53 expression in human lung cancer is associated with histologic subtypes and patient smoking history. *Am. J. Clin. Pathol.*, **102**, 660–664.

ESRIG D, ELMAJIAN D, GROSHEN S, FREEMAN JA, STEIN JP, CHEN S-C, NICHOLS PW, SKINNER DG, JONES PA AND COTE RJ. (1994). Accumulation of nuclear p53 and tumor progression in bladder cancer. *N. Engl. J. Med.*, **331**, 1259–1264.

GAZZERI S, BRAMBILLA E, CARON DE FROMENTEL C, GOUYER V, MORO D, PERRON P, BERGER F AND BRAMBILLA C. (1994). p53 genetic abnormalities and *myc* activation in human lung carcinoma. *Int. J. Cancer*, **58**, 24–32.

GRECO C, GANDOLFO GM, MATTEI F, GRADILONE A, ALVINO S, PASTORE LI, CASALE P, GRASSI A, CIANCIULLI AM AND AGLIANO AM. (1994). Detection of c-*myb* genetic alterations and mutant p53 serum protein in patients with benign and malignant colon lesions. *Anticancer Res.*, **14**, 1433–1440.

HARLOW E AND LANE D. (1988). *Antibodies: A Laboratory Manual*, pp. 53–287. Cold Spring Harbor Laboratory Press: Cold Spring Harbor, NY.

HASSAPOGLIDOU S, DIAMANDIS EP AND SUTHERLAND DJA. (1993). Quantification of p53 protein in tumour cell lines, breast tissue extracts and serum with time-resolved immunofluorometry. *Oncogene*, **8**, 1501–1509.

HOLLSTEIN M, SIDRANSKY D, VOGELSTEIN B AND HARRIS CC. (1991). p53 mutations in human cancers. *Science*, **253**, 49–53.

JOYPAUL BV, VOJTESEK B, NEWMAN EL, HOPWOOD D, GRANT A, LANE DP AND CUSCHIERI A. (1993). Enzyme-linked immunosorbent assay for p53 in gastrointestinal malignancy: comparison with immunohistochemistry. *Histopathology*, **23,** 465-470.

KUERBITZ SJ, PLUNKETT BS, WALSH WV AND KASTAN MB. (1992). Wild-type p53 is a cell cycle checkpoint determinant following irradiation. *Proc. Natl Acad. Sci. USA*, **50,** 379-384.

LAHDEAHO M-L, LEHTINEN T, AINE R, HAKALA T AND LEHTINEN M. (1994). Antibody response to adenovirus Elb-derived synthetic peptides and serum levels of p53 in patients with gastrointestinal and other malignant lymphomas. *J. Med. Virol.*, **43,** 393-396.

LEHTINEN T, AINE R, KELLOKUMPU-LEHTINEN P, HAKALA T AND LEHTINEN M. (1993). Evaluation of plasma levels of thymidine kinase and mutated p53 in 81 patients with newly diagnosed malignant lymphoma. *Acta Oncol.*, **32,** 779-781.

LEVESQUE MA, KATSAROS D, YU H, ZOLA P, SISMONDI P, GIARDINA G AND DIAMANDIS EP. (1995*a*). Mutant p53 protein overexpression is associated with poor outcome in patients with well or moderately differentiated ovarian carcinoma. *Cancer*, **75,** 1327-1338.

LEVESQUE MA, D'COSTA M, ANGELOPOULOU K AND DIAMANDIS EP. (1995*b*). Time-resolved immunofluorometric assay of p53 protein. *Clin. Chem.*, **41,** 1720-1729.

LEVESQUE MA, CLARK GM, YU H AND DIAMANDIS EP. (1995*c*). Immunofluorometric analysis of p53 protein and prostate specific antigen in breast tumours and their association with other prognostic indicators. *Br. J. Cancer*, **72,** 720-727.

LEVINE AJ, MOMAND J AND FINLAY CA. (1991). The p53 tumour suppressor gene. *Nature*, **351,** 453-456.

LOHMANN D, PUTZ B, REICH U, BOHM J, PRAUER H AND HOFLER H. (1993). Mutational spectrum of the p53 gene in human small-cell lung cancer and relationship to clinicopathologic data. *Am. J. Pathol.*, **142,** 907-915.

LOWE SW, SCHMITT EM, SMITH SW, OSBORNE BA AND JACKS T. (1993). p53 is required for radiation induced apoptosis in mouse thymocytes. *Nature*, **362,** 847-849.

LUBIN R, ZALCMAN G, BOUCHET L, TREDANIEL J, LEGROS Y, CAZALS D, HIRSCH A AND SOUSSI T. (1995). Serum p53 antibodies as early markers of lung cancer. *Nature Med.*, **1,** 701-702.

LUO J-C, ZEHAB R, ANTTILA S, RIDANPAA M, HUSGAFVEL-PURSIAINEN K, VAINIO H, CARNEY W, DEVIVO I, MILLING C AND BRANDT-RAUF PW. (1994). Detection of serum p53 protein in lung cancer patients. *J. Occup. Med.*, **36,** 155-160.

LUO-J-C, NEUGUT AI, GARBOWSKI G, FORDE KA, TREAT M, SMITH W, CARNEY WP AND BRANDT-RAUF PW. (1995). Level of p53 antigen in the plasma of patients with adenomas and carcinomas of the colon. *Cancer Lett.*, **91,** 235-240.

MAO L, HRUBAN RH, BOYLE JO, TOCKMAN M AND SIDRANSKY D. (1994). Detection of oncogene mutations in sputum precedes diagnosis of lung cancer. *Cancer Res.*, **54,** 1634-1637.

MARCHETTI A, BUTTITTA F, MERLO G, DIELLA PELLEGRINI S, PEPE S, MACCHIARINI P, CHELLA A, ANGELETTI CA, CALLAHAN R, BISTOCCHI M AND SQUARTINI F. (1993). p53 alterations in non-small cell lung cancers correlate with metastatic involvement of hilar and mediastinal lymph nodes. *Cancer Res.*, **53,** 2846-2851.

MIDGLEY CA, FISHER CJ, BARTEK J, VOJTESEK B, LANE DP AND BARNES BM. (1992). Analysis of p53 expression in human tumors: an antibody raised against human p53 expression in *Escherichia coli*. *J. Cell Sci.*, **101,** 183-189.

MILLER CW, SIMON K, ASLO A, KOK K, YOKOTA J, BUYS CHC, TERADA M AND KOEFFLER HP. (1992). p53 mutations in human lung cancers. *Cancer Res.*, **52,** 1695-1698.

MITSUDOMI T, OYAMA T, KUSANO T, OSAKI T, NAKANISHI R AND SHIRAKUSA T. (1993*a*). Mutations of the p53 gene as a predictor of poor prognosis in patients with non-small-cell lung cancer. *J. Natl Cancer Inst.*, **85,** 2018-2023.

MITSUDOMI T, LAM S, SHIRAKUSA T AND GAZDAR AF. (1993*b*). Detection and sequencing of p53 gene mutations in bronchial biopsy samples in patients with lung cancer. *Chest*, **104,** 362-365.

NAHM MH AND HOFFMANN JW. (1990). Heteroantibody: phantom of the immunoassay. *Clin. Chem.*, **36,** 829.

PAPPOT H, FRANCIS D, BRUNNER N, GRONDAHL-HANSEN J AND OSTERLIND K. (1996). p53 protein in non-small cell lung cancer as quantitated by enzyme-linked immunosorbent assay: relation to prognosis. *Clin. Cancer Res.*, **2,** 155-160.

PARTANEN P, KOSKINEN H, OKSA P, HEMMINKI K, CARNEY W, SMITH S AND BRANDT-RAUF P. (1995). Serum oncoproteins in asbestosis patients. *Clin. Chem.*, **41,** 1844-1847.

QUINLAN DC, DAVIDSON AG, SUMMERS CL, WARDEN WE AND DOSHI HM. (1992). Accumulation of p53 protein correlates with a poor prognosis in human lung cancer. *Cancer Res.*, **52,** 4828-4831.

REMVIKOS Y, TOMINAGA O, HAMMEL P, LAURENT-PUIG P, SALMAN RJ, DUTRILLAUX B AND THOMAS G. (1992). Increased p53 protein content of colorectal tumours correlates with poor survival. *Br. J. Cancer*, **66,** 758-764.

ROSANELLI GP, WIRNSBERGER GH, PURSTNER P AND STEINDORFER P. (1993). DNA flow cytometry and immunohistochemical demonstration of mutant p53 protein versus TPS and mutant p53 protein serum levels in human breast cancer. *Proc. Am. Assoc. Cancer Res.*, **34,** 227.

RYBERG D, KURE E, LYSTAD S, SKAUG V, STANGELAND L, MERCY I, BORRESEN A-L AND HAUGEN A. (1994). p53 mutations in lung tumors: relationships to putative susceptibility markers for cancer. *Cancer Res.*, **54,** 1551-1555.

SCHLICHTHOLZ B, TREDANIEL J, LUBIN R, ZALCMAN G, HIRSCH A AND SOUSSI T. (1994). Analyses of p53 antibodies in sera of patients with lung carcinoma define immunodominant regions in the p53 protein. *Br. J. Cancer*, **69,** 809-816.

SMITH ML, CHEN I-T, ZHAN Q, BAE I, CHEN C-Y, GILMER TM, KASTAN MB, O'CONNOR PM AND FORNACE AJ JR. (1994). Interaction of the p53-regulated protein Gadd45 with proliferating cell nuclear antigen. *Science*, **266,** 1376-1380.

SOUSSI T, LEGROS Y, LUBIN R, ORY K AND SCHLICHTHOLZ B. (1994). Multifactorial analysis of p53 alteration in human cancer: a review. *Int. J. Cancer*, **57,** 1-9.

SUZUKI H, TAKAHASHI T, KUROISHI T, SUYAMA M, ARIYOSHI Y, TAKAHASHI T AND UEDA R. (1992). p53 mutations in non-small cell lung cancer in Japan: associations between mutations and smoking. *Cancer Res.*, **52,** 734-736.

THOR AD, MOORE DH II, EDGERTON SM, KAWASAKI ES, REIHSAUS E, LYNCH HT, MARCUS JN, SCHWARTZ L, CHEN LC, MAYALL BH AND SMITH HS. (1992). Accumulation of p53 tumor suppressor gene protein: an independent marker of prognosis in breast cancers. *J. Natl Cancer Inst.*, **84,** 845-855.

TRUMPER L, JUNG W, DAHL DIEHL V, GAUSE A AND PFREUNDSCHUH M. (1994). Interleukin-7, interleukin-8, soluble TNF receptor, and p53 protein levels are elevated in the serum of patients with Hodgkin's disease. *Ann. Oncol.*, **5**(suppl.1), 93-96.

VOJTESEK B, BARTEK J, MIDGLEY CA AND LANE DP. (1992). An immunochemical analysis of human nuclear phosphoprotein p53: new monoclonal antibodies and epitope mapping using recombinant p53. *J. Immunol. Methods*, **151,** 237-244.

VOJTESEK B, FISHER CJ, BARNES DM AND LANE DP. (1993). Comparison between p53 staining in tissue sections and p53 protein levels measured by an ELISA technique. *Br. J. Cancer*, **67,** 1254-1258.

WALKER C, ROBERTSON LJ, MYSKOW MW, PENDLETON N AND DIXON GR. (1994). p53 expression in normal and dysplastic bronchial epithelium and in lung carcinomas. *Br. J. Cancer*, **70,** 297-303.

WINTER SF, MINNA JD, JOHNSON BE, TAKAHASHI T, GAZDAR AF AND CARBONE DP. (1992). Development of antibodies against p53 in lung cancer patients appear to be dependent on the type of p53 mutation. *Cancer Res.*, **52,** 4168-4174.

WORLD HEALTH ORGANIZATION. (1982). The World Health Organization. Histological typing of lung tumors. *Am. J. Clin. Pathol.*, **77,** 123-136.

ZUSMAN I, ZUSMAN R, KOROL D, SANDLER B, BEN-HUR H, BASS D, MASHIAH A, LIFSHITZ-MERCER B, SMIRNOFF P AND GLICK J. (1995). Isolation of p53 protein from the serum of colon cancer and non-cancer patients. *Oncol. Reports*, **2,** 679-683.

ZWEIG MH AND CAMPBELL G. (1993). Receiver-operating characteristic (ROC) plots: a fundamental evaluation tool in clinical medicine. *Clin. Chem.*, **39,** 561-577.